# Advances in the Treatment of Implant-Associated Infections of the Pelvis: Eradication Rates, Recurrence of Infection, and Outcome

**DOI:** 10.3390/jcm12082854

**Published:** 2023-04-13

**Authors:** Florian Kellermann, Simon Hackl, Iris Leister, Sven Hungerer, Matthias Militz, Fabian Stuby, Bernhard Holzmann, Jan Friederichs

**Affiliations:** 1 Trauma Center Murnau, Prof.-Küntscher-Str. 8, 82418 Murnau, Germany; 2 Department of Surgery, Klinikum Rechts der Isar München, 81675 Munich, Germany

**Keywords:** osteosynthesis, pelvic fractures, infection, eradication, recurrence

## Abstract

Introduction: Surgical site infections after operative stabilization of pelvic and acetabular fractures are rare but serious complications. The treatment of these infections involves additional surgical procedures, high health care costs, a prolonged stay, and often a worse outcome. In this study, we focused on the impact of the different causing bacteria, negative microbiological results with wound closure, and recurrence rates of patients with implant-associated infections after pelvic surgery. Material and Methods: We retrospectively analyzed a study group of 43 patients with microbiologically proven surgical site infections (SSI) after surgery of the pelvic ring or the acetabulum treated in our clinic between 2009 and 2019. Epidemiological data, injury pattern, surgical approach, and microbiological data were analyzed and correlated with long-term follow-up and recurrence of infection. Results: Almost two thirds of the patients presented with polymicrobial infections, with staphylococci being the most common causing agents. An average of 5.7 (±5.4) surgical procedures were performed until definitive wound closure. Negative microbiological swabs at time of wound closure were only achieved in 9 patients (21%). Long-term follow-up revealed a recurrence of infection in only seven patients (16%) with an average interval between revision surgery and recurrence of 4.7 months. There was no significant difference of recurrence rate for the groups of patients with positive/negative microbiology in the last operative revision (71% vs. 78%). A positive trend for a correlation with recurrent infection was only found for patients with a Morel–Lavallée lesion due to run-over injuries (30% vs. 5%). Identified causing bacteria did not influence the outcome and rate of recurrence. Conclusion: Recurrence rates after surgical revision of implant-associated infections of the pelvis and the acetabulum are low and neither the type of causing agent nor the microbiological status at the timepoint of wound closure has a significant impact on the recurrence rate.

## 1. Introduction

Unstable pelvic fractures usually result from a high-energy mechanism. Nonoperative treatment of such fractures often leads to significant disabilities. Therefore, various techniques for operative stabilization of both the anterior and posterior pelvic ring have been described [1,2,3]. However, due to extensive surgical approaches, the long duration of operative procedures, and concomitant soft-tissue damage and postoperative infection rates were reported to be as high as 18–27% in early series for posterior approaches of type C pelvic fractures [4] and have improved to rates below 5% in more recent studies [1]. Similar rates of infection have been reported after the osteosynthetic stabilization of the anterior pelvic ring and operative reconstruction of acetabular fractures [5,6]. Infection rates can be even higher, up to 50%, for open pelvic fractures or complex fractures with concomitant injuries of the rectum or bladder, resulting in worse overall outcomes [7,8].

A recent study by Karakaris et al. analyzed patients with deep infections following operative reconstruction of pelvic fractures and concluded that surgical site infections (SSI) are a rare but serious complication of pelvic surgery, occurring in 2.1% of cases. Injury- and surgery-related risk factors were identified, such as fracture type, high Injury Severity Score (ISS), long duration of surgery, and a posterior sacral approach. Significant patient factors included obesity, diabetes, and alcohol consumption [9]. Interestingly, no significant correlation was observed between surgical site infection and pelvic packing, pelvic arterial embolization (PAE), or Morel–Lavallee lesion, contrary to previous reports [10,11,12].

A surgical site infection of the pelvis can have serious consequences, such as prolonged hospital stay, increased healthcare costs, possible readmissions, and worse physical, social, and psychological outcomes [9,10]. Conservative treatment is not possible, and a long regimen of operations is required to eradicate the infection without compromising stability and function. Karakaris et al. found that up to 16 operations were necessary to achieve this aim, with a median number of 3 operations. However, complete eradication was achieved in 93% of patients [9].

While the prevention of surgical site infections has improved over the last decades due to advanced surgical techniques, identification of risk factors, and post-operative measures, only a few studies focus on the management and outcome of surgical site infections after pelvic surgery and little is known about treatment algorithms, effectiveness of different measures such as vacuum assistant closure (VAC), causative bacteria, negative microbiological results with wound closure, and long-term results after eradication [9]. There are several case reports and small series, for example, the recent study of Vaidya, where a series of 10 infections after anterior subcutaneous internal fixation of the pelvis were analyzed [13]. The predominant causative agent was *Staphylococcus aureus*; surgical irrigation and debridement, implant removal, and culture-specific antibiotics led to a favorable outcome in all ten patients. This goes in accordance to clinical practice, several case reports, and postoperative deep wound infections of other locations [10,11,12]. However, to our knowledge, there is no study that focuses on long-term recurrence rates of patients with posttraumatic infections of the pelvis.

Therefore, the aim of our study is to focus on the long-term results of patients with microbiologically proven surgical site infections after pelvic surgery. This rare subgroup of patients has not been previously studied, and no data exist regarding the impact of different causative bacteria, negative microbiological results with wound closure, and recurrence rates.

## 2. Material and Methods

The retrospective cohort single center study was conducted at our Level One Trauma Center. All patients with microbiologically proven surgical site infections (SSI) after surgery of the pelvic ring or the acetabulum treated in our clinic between 2009 and 2019 were included. The study adhered to ethical standards set by the institutional and national research committee and was approved by the local ethics committee in compliance with the 1964 Helsinki Declaration and its subsequent amendments. Patients provided written informed consent before receiving treatment. Exclusion criteria were patients aged <18 years, and patients with only soft-tissue infections or decubiti as well as infections after endoprosthetic surgery. The study recorded patient data such as sex, age, trauma mechanism, primary fracture classification according to the AO and Letournel systems, primary operative access, procedure, time to infection, number of operations, and length of hospital stay. It also documented the initial microbiological result, changes in detected causative bacteria, and microbiological result at the time of wound closure. Table 1 summarizes the patient data.

### 2.1. Surgical Procedure

All patients were treated in our Department of Septic Surgery following a standardized pre-, intra-, and post-operative management protocol. Preoperative management included a thorough clinical examination by the treating surgeon, a CT scan, an evaluation of comorbidities, a detailed analysis of the previous operative procedure, and a standardized blood analysis including all parameters of infection. The standardized intraoperative protocol of the index operation put the focus on the proof of the surgical site infection and the identification of the causing bacteria and thus was strictly followed by the operating surgeon. Surgery was performed under general anesthesia using pre-existent access if possible. Perioperative antibiotic treatment was initiated only after taking at least two swabs, and two pieces of tissue for microbiological and histological examination were taken from representative areas of the affected region. According to the protocol, the empirical antimicrobial regimen was continued until a modification according to the culture results was possible. In the index operation, hardware was removed only when an infection was macroscopically without doubt or had been proven before. If necessary, mechanical stability was restored by external fixation. The removal of the metalwork was followed by a radical debridement with resection of all fibrotic and macroscopically infected tissue of the interphase. After the administration of local antiseptic solution (Octenidin, Polyhexamide), vacuum-assisted closure (VAC) of the surgical site was achieved and a standardized multi-stage surgical revision protocol was started with operative debridement every 5–7 days based on clinical and biochemical parameters, the soft-tissue status, the extent of the infection, and on the virulence of the microorganism. This revision protocol was repeated until short-term cultures were negative, a macroscopically clean soft-tissue status was achieved and clinical and biochemical parameters had improved accordingly. The wound was then finally closed, and test-specific antimicrobial medication was continued for at least 6 weeks after the last surgical intervention.

### 2.2. Microbiological Examination

To conduct microbiological analysis, at least three dry swabs (MASTASWAB TM, Mast Group Ltd., Bootle, UK) were taken directly from the removed implant, the interface, and from macroscopically suspicious areas of the wound. The swabs were streaked out on Columbia agar with 5% sheep blood, chocolate agar, MacConkey agar, and thioglycolate broth (bioMerieux, Hazelwood, MO, USA). Samples were then incubated at 37 °C in 5% CO_2_ or anaerobic conditions for 48 h for short-term culturing; morphologically distinct colonies were identified and antibiotic susceptibility to 28 antibiotics was determined using the Vitek2-machine (bioMerieux, Hazelwood, MO, USA) with standardized definition of minimum inhibitory concentration (MIC) and multi-drug resistance [14]. At least two tissue samples from the interface, non-union, or macroscopically suspicious areas were directly inserted into a sterile containment prefilled with 9 ml of thioglycolate broth (bioMerieux, Hazelwood, MO, USA). After incubation at 37 °C in 5% CO_2_ or under anaerobic conditions for at least 14 days (long-term culturing), the suspension was additionally streaked out and proceeded as described above.

### 2.3. Follow-Up

Patients were followed up in our outpatient department at regular intervals after 6 weeks, 3 months, and 6 months. Follow-up included a clinical examination, systemic inflammatory parameters, and a radiological follow-up. Revision surgery and/or antibiotic treatment due to soft-tissue inflammation was documented. For long-term follow-up, patients were contacted via a short survey or by telephone focusing on recurrence of infection and conservative treatment or revision surgery due to recurrent infection. Loss of follow-up was documented if no contact with the patient was achieved.

### 2.4. Statistical Analysis

Statistical analysis was performed using IBM SPSS^®^Statistics for Windows 19.0 (IBM Corp., Armonk, NY, USA). Results of this study are presented as mean values ± standard error of the mean (SEM) or median. Significance was statistically calculated based on the Mann–Whitney U-test or Fisher’s exact test. Results were considered to be statistically significant with *p* values *<* 0.05.

## 3. Results

### 3.1. Epidemiology and Initial Surgical Approach

**Figure 1:**
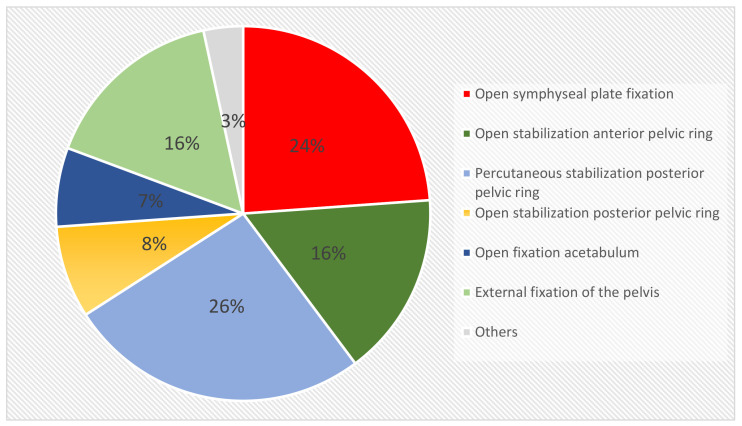
Initial surgical approaches of 43 patients with subsequent implant-associated infections after surgical treatment of fractures of the pelvis and the acetabulum (*n* = 88).

The study included 43 patients who had confirmed surgical site infections (SSI) following surgery of the pelvic ring or the acetabulum. The epidemiological information of the study participants is outlined in Table 1. Of the 43 patients, 27 (63%) received surgical stabilization of instable pelvic fractures (Type B (*n* = 11 (26%)) and C (*n* = 16 (37%)), 5 patients (12%) were treated for surgical site infections after isolated acetabular fractures, and, in 7 patients (16%), surgical intervention addressed the combination of unstable pelvic fracture and acetabular fracture. The remaining four patients comprised of two hemipelvectomies and two unclassified injuries. The injury patterns described above led to a total of 88 initial operative approaches involving the anterior (*n* = 37), the posterior (*n* = 43) pelvic ring, the acetabulum (*n* = 5), and others (*n* = 3), as shown in Figure 1.

### 3.2. Microbiology

**Figure 2:**
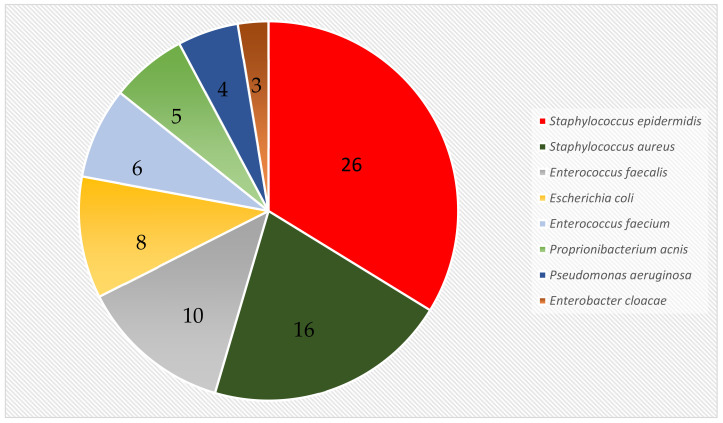
Microbiological results of 43 patients with implant-associated infections after surgical treatment of fractures of the pelvis and the acetabulum. Two thirds of infections were polymicrobial; 36 different bacteria and fungi were detected. The eight most frequent bacteria are listed.

The index operation aimed at the removal of all hardware, the identification of the causing agent, and a radical debridement of infected tissue. The identification of the causing pathogen was achieved in all 43 patients revealing a total of 36 different bacteria and fungi (Table S1). Over the course of revision surgery, *Staphylococcus epidermidis* was detected in 26 patients (60.5%) and *Staphylococcus aureus* in 16 patients (37.2%). The eight most frequent species are listed in Figure 2. Almost two thirds of the patients presented with polymicrobial infections (2–8 different bacteria and fungi); monomicrobial infections were observed in 14 patients (32.6%), half of them caused by *Staphylococcus epidermidis* (7 patients), four infections caused by *Staphylococcus aureus*, two by enterococci, and one by clostridium difficile. During the revision surgery, in 21 of 43 patients (48.8%), a change of the bacterial species was observed, while the intraoperative swabs showed a persistent colonization pattern throughout the surgical treatment in 22 patients (51.2%).

### 3.3. Eradication Rate and Recurrence of Infection

Revision surgery aimed at the eradication of the infection. However, an eradication with negative swabs at the time point of wound closure was only achieved for 9 patients (21%). A total of 34 wounds still had positive microbiological results in the long-term culture of the last operation (79%).

However, in the long-term follow-up, only seven patients (16%) suffered of a recurrent infection with an average time interval of 4.7 months between revision surgery and recurrence. Nine patients (21%) were lost to follow-up, while twenty-seven patients (63%) showed no signs of infection during a follow-up period ranging from 24 to 226 months. There was no significant correlation between recurrence rate and age, sex, surgical approach, fracture classification, type of osteosynthesis, number of surgical revisions, or early/late surgical site infection. Interestingly, there was no significant difference of recurrence rate for the groups of patients with positive/negative microbiology in the last operative revision (71% vs. 78%). A positive trend for a correlation with recurrent infection was only found for patients with a Morel–Lavallée lesion due to run-over injuries (30% vs. 5%). The identified bacteria did not influence outcome and rate of recurrence, the distribution of the most frequent germs was almost identical in both groups as shown in Table 2.

In summary, the likelihood of implant-associated infection recurrence in the pelvis and acetabulum following surgical revision is relatively low. The recurrence rate is not significantly affected by either the type of causative agent or the microbiological status at the time of wound closure.

## 4. Discussion

Surgical site infections following pelvic surgery are a rare yet severe complication that often require extended hospitalization, multiple revision surgeries, and prolonged antibiotic therapy. Proper management of soft tissues is crucial, but treating surgeons have noted a high rate of persistent infection and recurrence after revision surgery. Our study, which involved a large group of 43 patients, is the first to show that even after a long-term follow-up of two to nine years, the recurrence rate is relatively low at 16%, indicating a positive prognosis for revision surgery.

In most cases, revision surgery did not manage to completely eradicate the causing bacteria. Only in 9 patients (21%) negative microbiological results were achieved until secondary wound closure. However, the recurrence rate did not significantly differ between patients with a microbiologically eradicated site at the time of wound closure and those with persisting positive swabs. This finding is unexpected and could change the paradigm of postoperative infection treatment after pelvic surgery. Corresponding to our protocol, the eradication procedures of surgical site infections of the pelvis often involve multiple operations over a long period of time, putting a high burden on the patient. Advancements in surgical treatment with a more radical initial debridement in combination with the knowledge gained in our study could lead to fewer operations, shorter hospital stays, lower treatment costs, and less stress for the patient.

One of the largest studies on early reoperation of acetabular fractures due to surgical site infections was published by Ding and coworkers in 2018 [6]. Due to the large study collective, they were able to analyze 56 patients reoperated due to implant-associated infections after operative stabilization of acetabular fractures and reported an infection rate of 7% which is comparable to other reported infection rates after acetabular surgery [15,16,17]. The median time for postoperative infection occurred at 2.4 weeks after the index operation, with a range of up to 102 weeks. Presumably, the rate of early and late infections is comparable to our study collective where 56% of infections were early infections (<6 weeks). In contrast to our study, microbiological examination proved polymicrobial infections in only approximately one third of the patients while we were able to identify more than one causing agent (up to eight) in two thirds of the patients. Nevertheless, the genus distribution was comparable to our study and other reported results with *Staphylococcus aureus*, *Staphylococcus epidermidis*, and *Enterococcus faecalis* being the most common bacteria [16,17]. In contrast, Torbert et al. detected a higher percentage of gram-negative bacteria with up to 63% of all infections [18]. Although many of the prevailing studies describe the standard surgical procedure for the treatment of deep surgical site infections of the pelvis, there is little information about the success of these operations. In their study, Suzuki and coworkers describe a mean of 3.3 surgical revisions for a deep infection with a range of 1–13 operations [17]. Only 40% of the cases necessitated implant removal, and culture specific local and systemic antibiotic therapy was administered according to international standards. However, no information regarding long-term success and recurrence rates is provided.

The Morel–Lavallée lesion is described as an internal degloving injury caused by shear forces on the soft tissue of the pelvis, a frequent concomitant injury of severe pelvic fractures. Due to the severity of the fracture and of other concomitant injuries, the Morel–Lavallée lesion is often underestimated and undertreated. Several studies have proven that the risk of soft-tissue infection of this lesion is high [17,19,20] and the risk of a surgical sight infection even on other locations of the pelvis is increased [17,21]. Our study adds the important results, that the recurrence rate after revision surgery of implant-associated infections of the pelvis is higher in patients with an initial Morel–Lavallée lesion. This finding is significant and should be considered when determining the appropriate surgical treatment for infections in the pelvis.

There are certain limitations of this retrospective single center cohort study. The surgical treatment in a single center might lead to a selection bias; the heterogeneous operative approaches might also act as confounding factors. However, the series of 43 patients operatively revised for implant-associated infections of the pelvis is the largest series found in the literature.

## 5. Conclusions

In summary, our study’s findings may contribute to advancements in surgical treatments for implant-associated infections following pelvic surgery. Contrary to the belief that these infections are difficult to treat and have a poor prognosis with a high recurrence rate, our long-term follow-up showed a recurrence in only 16% of patients. Furthermore, our data suggests that performing one or more negative swabs prior to wound closure may not be necessary for successful therapy, as recurrence rates were similar for both negative and positive wound closures. This implies that fewer operations may be required to achieve treatment success. Nevertheless, we recommend thorough soft-tissue management and potentially more aggressive surgical debridement for patients with previous soft-tissue problems such as the Morel–Lavallée lesion.

## Figures and Tables

**Table 1:** Clinical characteristics of 43 patients with implant-associated infections of the pelvis and the acetabulum.

Age (years)	45.4 (±15.4)
Male	32 (74.4%)
Female	11 (25.6%)
Early infection (<6 weeks)	24 (56%)
Late infection >6 weeks)	19 (44%)
Days in hospital (median)	45 (7–330)
Number of operations (average)	5.7 (±5.4)
Follow-up (median, months)	98.2 (24–226)

**Table 2:** Most frequent causing bacteria of implant-associated infections after pelvic surgery related to recurrence of infection in long-term follow-up. Note that polymicrobial infections were found in almost two thirds of the patients.

Recurrent Infection	Non-Recurrent Infection
*n* = 7 (16%)	27 (63%)
*Staphylococcus epidermidis* (57%)	*Staphylococcus epidermidis* (67%)
*Enterococcus faecalis* (43%)	*Staphylococcus aureus* (39%)
*Staphylococcus aureus* (29%)	*Enterococcus faecalis* (19%)
	*Escherichia coli* (19%)
	*Enterococcus faecium* (17%)
	*Pseudomonas aeruginosa* (8%)

## Data Availability

Data are not available due to privacy and ethical restrictions.

## Supplementary Materials

The following supporting information can be downloaded at: https://www.mdpi.com/article/10.3390/jcm12082854/s1, Table S1. All detected bacteria and fungi, the first eight bacterial were detected in twice, all other bacteria and fungi were only detected once.
