# Supplementary material for: Advances in the Treatment of Implant-Associated Infections of the Pelvis: Eradication Rates, Recurrence of Infection, and Outcome
Source: J Clin Med. 2023 Apr 13;12(8):2854. doi: 10.3390/jcm12082854 (PMC10145122; doi:10.3390/jcm12082854)
Supplement: Supplementary file 1 [file jcm-12-02854-s001.zip › jcm-2293701-supplementary.pdf]

Table S1. All detected bacteria and fungi, the first eight bacterial were detected in twice, all other bacteria and fungi were only detected once.

**Bacteria**
Citrobacter coseri
Klebsiella pneumoniae
Peptostreptococcus species
Proprioni species
Proteus vulgaris
Staphylococcus haemolyticus
Staphylococcus lugdunensis
Staphylococcus warneri
Bacteroides fragilis
Bacteroides thetaiotaomicron
Candida albicans
Candida glabrata
Citrobacter freundii
Clostridium difficile
Corynebacterium species
Enterobacter amnigenus
Enterobacter hormaechei
Finegoldia magna
Klebsiella oxytoca
Morganella morganii
Peptostreptococcus prevotii
Providencia vulgaris
Serratia
Staphylococcus capitis
Streptococcus agalactiae
Streptococcus mitis
Streptococcus parasanguinis
Streptococcus salivarius
